# Psychometric Properties of the Internet Gaming Disorder Scale–Short-Form (IGDS9-SF): Systematic Review

**DOI:** 10.2196/26821

**Published:** 2021-10-18

**Authors:** Lok Y J Poon, Hector W H Tsang, Tsan Y J Chan, Sze W T Man, Lok Y Ng, Yi L E Wong, Chung-Ying Lin, Chi-Wen Chien, Mark D Griffiths, Halley M Pontes, Amir H Pakpour

**Affiliations:** 1 Department of Rehabilitation Sciences The Hong Kong Polytechnic University Hung Hom Hong Kong; 2 Mental Health Research Centre The Hong Kong Polytechnic University Hung Hom Hong Kong; 3 Institute of Allied Health Sciences College of Medicine National Cheng Kung University Tainan Taiwan; 4 Department of Public Health National Cheng Kung University Hospital, College of Medicine National Cheng Kung University Tainan Taiwan; 5 Department of Occupational Therapy College of Medicine National Cheng Kung University Tainan Taiwan; 6 Biostatistics Consulting Center National Cheng Kung University Hospital, College of Medicine National Cheng Kung University Tainan Taiwan; 7 International Gaming Research Unit Department of Psychology Nottingham Trent University Nottingham United Kingdom; 8 Department of Organizational Psychology Birkbeck University of London London United Kingdom; 9 Department of Nursing School of Health and Welfare Jönköping University Jönköping Sweden

**Keywords:** psychometrics, IGDS9-SF, gaming addiction, gaming disorder, problematic gaming, internet, gaming, internet gaming

## Abstract

**Background:**

The Internet Gaming Disorder Scale–Short-Form (IGDS9-SF) is among the best with regard to its psychometric properties. Therefore, clinical psychologists are likely guided to use the IGDS9-SF if they want to assess or screen the disordered gaming in their practice. However, the information, especially psychometric evidence, concerning the IGDS9-SF has not been fully examined and summarized.

**Objective:**

This systematic review evaluated the psychometric properties of different language versions of the IGDS9-SF and assessed its methodological quality in order to improve the clinicians’ understanding of the IGDS9-SF and facilitate its use.

**Methods:**

Systematic literature searches were carried out using *Embase*, *MEDLINE*, *PsycINFO*, *PubMed*, *ScienceDirect*, *Scopus*, and *Web of Science*. The review included English-language studies of any research design that have reported at least one psychometric property of the IGDS9-SF, as defined by the COnsensus-based Standards for the selection of health status Measurement INstrument (COSMIN), and have aimed at testing the psychometric properties of the IGDS9-SF.

**Results:**

In total, 21 studies comprising 15 language versions of the IGDS9-SF were included. Overall, the IGDS9-SF showed adequate internal consistency (although some items did not have satisfactory item-total correlation [IT]), excellent criterion validity, and the ability to distinguish different subgroups with measurement invariance being supported across gender and age. In terms of factor structure, the IGDS9-SF was shown to have a unidimensional factor structure across all 21 studies.

**Conclusions:**

Although there is insufficient evidence regarding the responsiveness and properties of the IGDS9-SF using item response theory, the existing evidence supports its use in assessing disordered gaming among individuals.

## Introduction

In the era of technology, internet use has become one of the essential components of everyone’s life [1]. However, internet use can be a potential hazardous tool for a minority of individuals, particularly adolescents and emerging adults [2]. Many websites and mobile applications, including online games, provide highly interactive features and services (eg, forums) that attract millions of users worldwide. Users, especially younger people, may therefore be unable to fully control their internet use as they encounter challenges to their self-control, alongside the addictive features of these applications, which tend to be associated with poor self-control levels among dysregulated and disordered users [3,4]. Therefore, they may end up spending a substantial amount of time within different virtual environments because they want to engage in social interaction on the internet [2]. In other words, internet use (such as online communication and gaming) may be a facilitator of reinforcing social relationships due to peoples’ preference for online social interaction [5].

Despite the many positive outcomes associated with online gaming, such as decreased loneliness and promotion of psychological well-being [6], the negative and dysfunctional effects of online gaming were observed in a minority of individuals in a systematic review and meta-analysis (N=226,247 from 53 studies across 17 countries, including European, American, and Asian countries, with different populations, eg, adolescents, gamers, and the general population, with a prevalence of 3.05% with a 95% confidence interval between 2.38% and 3.91%) [7]. Its negative impacts on both physical and mental health (eg, poor sleep quality, musculoskeletal discomfort, and increased psychological distress) have been widely reported in recent years [8,9]. Consequently, internet gaming disorder (IGD) was incorporated into the fifth revision of the American Psychiatric Association’s (APA) *Diagnostic and Statistical Manual of Mental Disorders* (DSM-5) as a tentative disorder and behavioral addiction [10].

Additionally, the 11th revision of the *International Classification of Diseases* (ICD-11) also formally recognized IGD and categorized it as an addictive disorder [11]. However, diagnosis in the ICD-11 relies upon clinical and functional impairment by manifesting pathological aspects rather than biological concepts, such as withdrawal and tolerance, as suggested in the DSM-5 in relation to IGD [11].

Ever since the emergence of the first internet addiction scale in 1998 [12], many psychometric assessment tools assessing the addictive effects of problematic internet use have been developed. More recently, self-report psychometric tests assessing IGD have been developed to assess the following nine IGD criteria proposed in the DSM-5 [10]: (1) preoccupation or obsession with gaming; (2) withdrawal symptoms when unable to engage in gaming; (3) tolerance, leading to necessity of spending more time in gaming for satisfying the urge of gaming; (4) inability to control participation in gaming; (5) not engaging in other hobbies and entertainment because of gaming; (6) persisting in excessive gaming irrespective of the psychosocial problems; (7) lying to family members or others in respect to the time spent on gaming; (8) using gaming to escape negative mood states; and (9) compromising occupation/education/significant relationships due to the involvement in gaming [13].

Currently, there are at least seven psychometric assessment tools for IGD that have been developed using DSM-5 criteria [14-17]. Among these seven instruments, two instruments rate responses using a Likert-type scale, two instruments rate items using either a Likert-type scale or a dichotomous scale, and one instrument begins rating items with a Likert-type scale and then converts to a dichotomous scale. More specifically, the 20-item Internet Gaming Disorder Test (IGDT-20) and the 9-item Internet Gaming Disorder Scale–Short-Form (IGDS9-SF) both use a 5-point Likert scale from 1 (never) to 5 (very often) and use the scale for scoring [18,19]. The 10-item Internet Gaming Disorder Test (IGDT-10) uses a 3-point Likert scale (ie, 0=never, 1=sometimes, and 2=often), which is then converted to a dichotomous score (ie, 0=never, 1=sometimes and often) [10]. The 27-item Internet Gaming Disorder Scale (IGDS) [20] and the IGDS9-SF [18] use either a 6-point Likert scale from 0 (never) to 5 (every day or almost every day) or a dichotomous scale (0=no and 1=yes).

Among the aforementioned psychometric instruments, the IGDS9-SF emerges as a robust and concise psychometric tool for assessing individuals with IGD, with a recent study identifying this tool as having great support in relation to its sound psychometric properties and significant advantages in comparison to most existing tools for IGD [21]. The IGDS9-SF includes all nine IGD criteria proposed by the APA in the DSM-5, with the features of conciseness and brief administration time, which is of great pragmatic utility in busy clinical settings when screening for the risk of IGD. Moreover, the psychometric properties of the IGDS9-SF have been widely assessed, including structural validity, internal consistency, cross-cultural validity/measurement invariance, reliability, measurement error, criterion validity, convergent validity, and discriminative or known-group validity.

In the current literature, the IGDS9-SF has been translated into 17 languages: Chinese, with three sublanguages of traditional Chinese in Hong Kong [22-24], traditional Chinese in Taiwan [22-24], and simplified Chinese in mainland China [25]; Albanian [26]; Italian [26]; English [26]; European and South American Portuguese [27,28]; Slovenian [29]; Persian [30]; Polish [31]; Spanish [32-34]; Turkish [35]; German [36]; Czech [37]; Malay [38]; and Korean [39]. As a screening tool, the IGDS9-SF can help clinicians in assessing IGD severity and the detrimental health impacts on the individual’s life with reasonable accuracy in a time-efficient way. Therefore, it has increasingly been psychometrically examined and used widely in epidemiological studies [40].

Although the psychometric properties of the IGDS9-SF have been examined among different populations, to the best of our knowledge, there is no systematic review of the IGDS9-SF reporting its psychometric characteristics in depth. Dispersed information in the extant literature regarding the IGDS9-SF with varying sample sizes and across different countries makes it timely to investigate whether clinicians should be adopting the IGDS9-SF for assessment of IGD. More specifically, there are a number of questions that are best answered by carrying out a systematic review of the psychometric properties of the IGDS9-SF.

First, the IGDS9-SF may have different psychometric features across different language versions, and it is unclear whether the existing psychometric evidence for different language versions of the IGDS9-SF is equivalent. Second, prior psychometric testing studies on the IGDS9-SF need to be evaluated for their methodological quality.

Without formal assessment of the quality of previous studies on the IGDS9-SF, the results pertaining to its psychometric properties may be biased. For the sake of improving clinicians’ understanding and facilitating the use of IGDS9-SF across other contexts beyond research settings, this systematic review incorporated different items of evidence concerning the psychometric features of the IGDS9-SF across a wide range of populations. More specifically, if the psychometric properties of the IGDS9-SF are found to be supported across different contexts, clinicians can use the IGDS9-SF criteria to exchange their expert opinions using the same signs, symptoms, and components. For example, a clinician in Taiwan can use IGDS9-SF scores to assess the IGD severity level of a disordered gamer, and this information can be well understood and correctly interpreted by clinicians in other countries.

Furthermore, when carrying out psychological assessment, clinicians are required by professional governing bodies (eg, the American Psychological Association, the British Psychological Society, the Australian Psychological Society) to adopt valid and reliable psychometric tools to support the adoption of evidence-based practices. Therefore, this review is of importance to clinicians working with disordered gamers.

## Methods

This review followed the recommended flow of the Preferred Reporting Items of Systematic Reviews and Meta-analyses (PRISMA) guidelines [41]. The evaluation of the psychometric properties of the IGDS9-SF was conducted with reference to the COnsensus-based Standards for the selection of health status Measurement INstrument (COSMIN) guidelines for systematic reviews of patient-reported outcome measures (PROMs) [42]. The review protocol was registered in the international prospective register of systematic reviews (PROSPERO; registration no. CRD42020198376).

### Search Strategy

A literature search was carried out using *Embase*, *MEDLINE*, *PsycINFO*, *PubMed*, *ScienceDirect*, *Scopus*, and *Web of Science* (these seven databases were used because they are commonly used databases in this field of psychology) to retrieve relevant studies published up to March 31, 2020, with the following search strategy: IGDS9-SF [All fields] OR IGDS-SF9 [All fields] OR IGD-SF [All fields] OR Internet Gaming Disorder Scale-Short-form [All fields] OR Internet Gaming Disorder Scale Short form [All fields] OR Internet Gaming Disorder Scale - 9-Item Short Form [All fields] OR 9-item Internet Gaming Disorder Scale - Short Form [All fields]. Slight modifications were made to the search strategy in order to optimize the search within each database (Multimedia Appendix 1).

A total of 2533 journal articles were identified. Duplicates (n=200) were removed using EndNote. The titles and abstracts of the remaining journal articles (n=2333) were screened for eligibility by two authors independently (ie, the same two authors who screened all 2333 journal articles). Of these, 2286 articles did not focus on the IGDS9-SF and were removed. Full texts of all potential articles were then retrieved (n=47) and screened using the same procedure. Of these, 26 were removed because of the following reasons: (1) the study did not test the psychometric properties of the IGDS9-SF (n=22), (2) the study was a non-peer-reviewed conference paper (n=3), or (3) the study was a review paper (n=1). The remaining 21 studies were evaluated and analyzed in this systematic review.

### Study Selection

The review included only English-language studies of all types of research design under the condition that they (1) reported at least one psychometric property of the IGDS9-SF (eg, internal consistency, reliability, measurement error, content validity, construct validity, criterion validity, or responsiveness), as defined by COSMIN, and (ii) aimed at testing the psychometric properties of the IGDS9-SF. Exclusion criteria were nonrefereed studies, review studies, conference proceedings, dissertations, commentaries, editorials, or letters to journal editors. The aforementioned inclusion and exclusion criteria were applied to all paper titles and abstracts, and screening of full texts.

### Evaluation of Psychometric Properties

The psychometric properties of the included studies were evaluated by five authors (TYJC, SWTM, LYN, LYJP, and YLEW), and each study was independently assessed by any two of the five authors using the COSMIN Risk-of-Bias checklist [43] according to the user manual of the COSMIN methodology for systematic reviews of PROMs. Following this, the corresponding author (C-YL) verified the evaluation results made by the five authors. The checklist comprised eight assessment properties: structural validity (ie, the property assessing whether the IGDS9-SF has a unidimensional structure), internal consistency (ie, the property assessing whether the nine items in the IGDS9-SF assess the same underlying construct, ie, IGD), cross-cultural validity/measurement invariance (ie, the property assessing whether the IGDS9-SF is interpreted similarly across different subgroups/cultures), reliability (ie, the property assessing whether the IGDS9-SF can be reproduced), measurement error (ie, the property reporting the error that cannot be assessed using the IGDS9-SF), criterion validity (ie, the property assessing the association between the IGDS9-SF and the gold standard assessing the same construct of IGD), hypothesis testing for construct validity (ie, the property assessing the association between the IGDS9-SF and a tool assessing a similar construct of IGD [ie, concurrent validity] or a tool assessing a construct different from IGD [ie, discriminant validity]), and responsiveness (ie, the property assessing whether the IGDS9-SF can effectively detect the improvement of IGD when effective treatment is given). It was noted that ΔCFI of >–0.01 would be used for evaluation of measurement invariance (where CFI is the comparative fit index) [44]. In the evaluation of criterion, concurrent, and convergent validity, coefficient values greater than or equal to 0.5 indicated strong correlation, values between 0.3 and 0.5 indicated fair correlation, and values between 0.1 and 0.3 indicated poor correlation [45].

Ranging from 3 to 13 items, each property was scored on a 4-point scale with four predefined options: very good (V), adequate (A), doubtful (D), and inadequate (I) [43]. The overall score of a psychometric property was graded based on the worst-score-counts principle [43]. Psychometric properties that were not available in the published study were marked as not applicable (NA). In this review, structural validity, internal consistency, cross-cultural validity/measurement invariance, reliability, measurement error, criterion validity, concurrent validity, convergent validity, and discriminant validity were synthesized, evaluated, and reported. Furthermore, additional psychometric properties, including the floor and ceiling effects and item-total correlation (IT), were also reported.

### Assessments of the Quality of Statistical Findings

The quality of the statistical results of each reported measurement property of the IGDS9-SF was rated against the updated criteria for good measurement properties based on Terwee et al [46] and Prinsen et al [47]. Each criterion was rated as sufficient (+), insufficient (–), or indeterminate (?), with each result being compared against the criterion and reported in the results table.

## Results

### Study Selection

**Figure 1:**
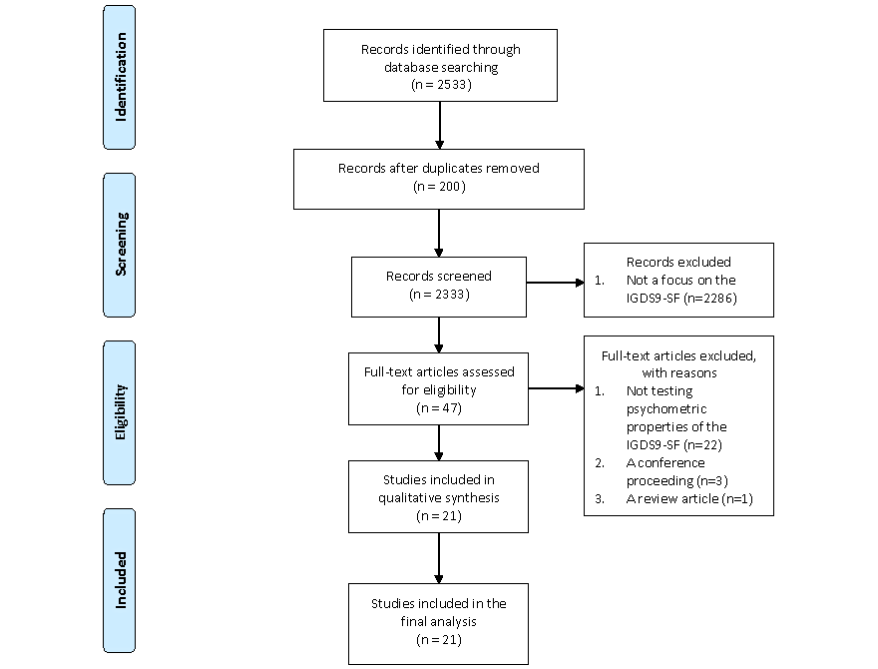
Flowchart of study selection [41]. IGDS9-SF: Internet Gaming Disorder Scale–Short-Form.

Of the 2533 identified studies, 200 were duplicates and 2333 were screened for abstracts (Figure 1). A total of 47 studies met the inclusion criteria and underwent subsequent full-text screening, of which 26 studies were further excluded due to the following reasons: the focus was not on the IGDS9-SF (n=22), they were conference proceedings (n=3), or they were review studies that compared different instruments without detailed information and quality assessment of each instrument (n=1). Therefore, a total of 21 studies on 15 language versions of the IGDS9-SF (ie, English, European Portuguese, South American Portuguese, Spanish, Albanian, Italian, Turkish, Slovenian, Polish, Persian, Malay, Korean, and Chinese, with three sublanguages of traditional Chinese in Hong Kong, traditional Chinese in Taiwan, and simplified Chinese in mainland China) were found to be relevant and included in the qualitative synthesis.

### Demographic Features

In terms of sample characteristics across all eligible studies, nine studies included gamers [19,26,31,38,39,48-51], two studies included people from gaming halls [26,52], one study comprised e-sports tournament (Electronic Sports League [ESL]) amateur e-sport players [48], and two studies included adults from the general community who played massively multiplayer online (MMO) games [38,51]. In addition, nine studies included university students [22-24,26,28,35,39,48,52,53], two studies included high school students [28,30], and two studies included students without specifying the education level [26,52]. Furthermore, one study included students learning English in a private institution and students in a private teaching institution [36]. The remaining studies included students in the sixth to ninth grades [29], students in the eighth grade [29], primary students in the fourth to sixth grades [24], and vocational training students [31]. The characteristics and demographics of the included studies are summarized in Table 1. Although these studies recruited participants with different demographic characteristics, the findings on the properties of the IGDS9-SF were similar, and the psychometric properties are summarized in Table 2. Detailed information about the psychometric properties is reported descriptively in subsequent sections.

**Table 1:** Characteristics of included studies and psychometric properties of the IGDS9-SF^a^ not included in COSMIN^b^.

Author/country	Study design	Population	Sample size	Age (years)	Testing period	Others (not included in COSMIN)
Chen et al [24]/Hong Kong and Taiwan	Cross-sectional	University students	Hong Kong 304 (67.4% female) Taiwan 336 (50.3% female)	Hong Kong 24.18 ± 4.92 Taiwan 20.51 ± 1.22	3 months (for testing temporal invariance)	Skewness=0.21-2.41 Kurtosis=−1.06-6.74
Beranuy et al [32]/Spain	Cross-sectional	Vocational training students	535 (21.5% female)	18.35 ± 2.13	N/A^c^	IT^d^=0.47-0.67
de Palo et al [26]/Albania, USA, UK, and Italy	Cross-sectional	Albania and Italy: People from schools, universities, and gaming halls USA and UK: English-speaking gamers from popular online gaming forums	Overall 1411 (36.4% female) Albania 228 (50.9% female) USA 237 (21.7% female) UK 275 (13.9% female) Italy 671 (45.4% female)	Overall 25.94 ± 8.91 Albania 31.38 ± 10.97 USA 29.09 ± 10.72 UK 29.50 ± 9.48 Italy 21.62 ± 3.9	N/A	No item showed absolute values of skewness >2 or values of kurtosis >7
Evren et al [48]/Turkey	Cross-sectional	University students, active internet gamers, and ESL Turkey amateur e-sport players	457 (62.4% female)	N/A	N/A	IT=0.663-0.826
Gomez et al [49]/USA	Cross-sectional	Internet gamers	868 (39.7% female)	Overall 28.64 ± 8.79 Male 27.94 ± 7.95 Female 20.09 ± 9.29	N/A	N/A
Leung et al [22]/Taiwan and Hong Kong	Cross-sectional	University students	Hong Kong 306 (67.6% female) Taiwan 336 (50.3% female)	Hong Kong 24.08 ± 5.06 Taiwan 20.51 ± 1.22	N/A	IT=0.68-0.85
Monacis et al [52]/Italy	Cross-sectional	Students from Italian schools, universities, and gaming hall	687 (45.4% female)	21.62 ± 3.90	N/A	No item showed absolute values of skewness >2 or values of kurtosis >7
Pontes and Griffiths [19]/English-speaking countries	Cross-sectional	English-speaking gamers from 58 different countries	1060 (14.9% female)	27 ± 9.02	N/A	Floor effect=3.8%-5.6% Ceiling effect=0.2%-0.8%
Pontes and Griffiths [27]/Portugal	Cross-sectional	Students in sixth, seventh, eighth, and ninth grades of a major located in the Algarve	509 (47.9% female)	13 ± 1.64	N/A	No item showed absolute values of skewness >3 or values of kurtosis >9
Pontes et al [50]/USA, India, and UK	Cross-sectional	English-speaking gamers from English-speaking online gaming forums that are popular among gamers	USA 405 (38% female) India 336 (32.4% female) UK 272 (49.3% female)	USA 32.57 ± 11.33 India 30.37 ± 8.90 UK 41.61 ± 14.03	N/A	N/A
Pontes et al [29]/Slovenia	Cross-sectional	Students in eighth grade	1071 (49.8% female)	13.44 ± 0.59	N/A	No item showed absolute values of skewness >3 or values of kurtosis >9
Schivinski et al [31]/Poland	Cross-sectional	Gamers	3377 (17.4% female)	20 ± 4.3	N/A	Skewness=–0.08-1.51 Kurtosis=–0.91-1.19
Severo et al [28]/Brazil	Cross-sectional	High school and college students	555 (42.5% female)	20.3 ± 5.4	N/A	No item showed absolute values of skewness >3 or values of kurtosis >9 IT=0.342-0.668
Stavropoulos et al [54]/USA and Australia	Longitudinal	Emerging adults from the general community who played massively multiplayer online (MMO) games	Australia 61 (26.2% female) USA 120 (40.2% female)	Australia 22.53 ± 3.04 USA 22.35 ± 2.82	60-90 days	N/A
Wu et al [30]/Iran	Cross-sectional	High school adolescents	2363 (35.2% female)	15.6 ± 1.2	2 weeks (for testing test-retest reliability)	IT=0.54-0.74 Floor effect=0.8% Ceiling effect=1.8%
Yam et al [23]/Hong Kong	Cross-sectional	University students	307 (67.6% female)	21.64 ± 8.11	N/A	IT=0.527-0.724 Floor effect=21% Ceiling effect=0%
Stavropoulos et al [51]/Australia, USA, and UK	Cross-sectional	Internet gamers	Australia 171 (23.4% female) USA 463 (42.1% female) UK 281 (13.9% female) Total 915 (55.1 female)	Australia 25.72 ± 5.52 USA 25.23 ± 2.76 UK 29.49 ± 9.47 Total 15.54 ± 0.65	N/A	N/A
Arıcak et al [35]/Turkey	Cross-sectional	Group 1: Students learning in English in a private institution Group 2: University students learning in English Group 3: Students from fifth grade to final year of university Group 4: Students in a private teaching institution	Group 1, 35 (54% female) Group 2, 33 (42% female) Group 3, 455 (46% female) Group 4, 64 (48% female)	Group 1, 12.50 ± 1.20 Group 2, 23.94 ± 1.52 Group 3, 15.83 ± 4.16 Group 4, 13.84 ± 1.59	Group 4: 2 weeks (for testing test- retest reliability)	Floor effect=9% Ceiling effect=0%
Chen et al [25]/mainland China	Cross-sectional	Primary school children in fourth to sixth grades	1108 (51.7% female)	10.37 ± 0.95	N/A	IT=0.55-0.76 Floor effect=24.6% Ceiling effect=0%
Kim and Ko [39]/Korea	Cross-sectional	Korean internet game users from major online gaming forums, universities, counseling centers, and libraries located in the greater Seoul area, Gyeonggi and Chungcheong Provinces of Korea	594 (29.6% female)	23.5 ± 6.29	N/A	IT=0.49-0.68
T’ng and Pau [38]/Malaysia	Cross-sectional	Youth who played MOBA^e^	1050 (25.1% female)	21.96 ± 2.37	N/A	No item showed absolute values of skewness >2 or values of kurtosis >7 Floor effect=0.1% Ceiling effect=1.7%

^a^IGDS9-SF: Internet Gaming Disorder Scale–Short-Form.

^b^COSMIN: COnsensus-based Standards for the selection of health status Measurement Instrument.

^c^N/A: not available.

^d^IT: item-total correlation

^e^MOBA: multiplayer online battle arena

**Table 2:** Summarized psychometric properties of the IGDS9-SF^a^ in the analyzed studies.

Psychometric property included in COSMIN^b^	N	Meth qual^c^	Result (rating^d^)
Structural validity	19,049	I	One factor (+)
Internal consistency	19,049	V	.810-.963 (+)
Cross-cultural validity	7352	I	Age (+) full invariance Gender (?) partial invariance Time on gaming (+) partial invariance Country (–) partial invariance
Reliability	2962	D	ICC^e^=.94 Pearson correlation=.756-.87
Measurement error	2962	D	0.16-2.27 (?)
Criterion validity	457	V	r=.988 (+)
Concurrent validity	12,323	V	Absolute r=.00-.556 Absolute β=.103-.663
Convergent validity	6149	V	Absolute r=.06-.827
Discriminative validity	1142	D	Significant difference found in age and gender

^a^IGDS9-SF: Internet Gaming Disorder Scale–Short-Form.

^b^COSMIN: Consensus-based Standards for the selection of health status Measurement Instrument.

^c^COSMIN score after removing the sample size item from the rating: V, very good; A, adequate; D, doubtful; I, inadequate; N, not applicable.

^d^Quality score of the measurement property: +, sufficient; -, insufficient; ?, indeterminate.

^e^ICC: intraclass correlation coefficient.

### Structural Validity

All studies reported the structural validity of the IGDS9-SF for the 15 versions (n=19,049). Of the 21 studies, 14 demonstrated very good methodological quality and had a positive rating for the quality of statistical findings; 6 studies with good to excellent methodological quality showed an indeterminate rating on the quality of statistical findings due to the absence of the standardized root-mean-square residual (SRMR; Supplementary Table S1 in Multimedia Appendix 2); and 1 study related to the Turkish version and with poor methodological quality had an indeterminate rating on the quality of statistical findings of structural validity (CFI=.987, root-mean-square error of approximation [RMSEA]=.064).

In addition, one study performed Rasch analysis to test the person separation reliability, person separation index, item separation reliability, and item separation index of the Persian version of the IGDS9-SF. The results showed doubtful methodological quality and a positive rating for the quality of statistical findings (person separation reliability=.86, person separation index=2.50, item separation reliability=1.00, item separation index=28.79). The study also reported an acceptable range of infit (0.79-1.37) and outfit mean square (0.74-1.34), as well as the range of item difficulties (–1.06-1.57).

### Internal Consistency

All studies evaluated the internal consistency of the IGDS9-SF for the 15 versions (n=19,049). All studies had very good methodological quality and showed a positive rating for the quality of statistical findings concerning internal consistency (Cronbach α=.810-.963 and person separation reliability=.86; see Supplementary Table S1 in Multimedia Appendix 2).

### Cross-Cultural Validity/Measurement Invariance

Of the 21 studies, 9 examined the measurement invariance of the IGDS9-SF across different factors (n=7352; see Supplementary Table S2 in Multimedia Appendix 2). The measurement invariance across age and gender was found to be fully or partially supported in these studies. Furthermore, one study on the Persian version found that partial invariance is supported across hours spent online gaming per week.

In addition, four studies reported the measurement invariance across multiple countries, and one study supported full invariance across the traditional Chinese (Hong Kong and Taiwan) versions. Partial metric and scalar invariance were supported across Albania, the United States, the United Kingdom, and Italy but neither across the United States, the United Kingdom, and India nor across the United States, the United Kingdom, and Australia.

Furthermore, one study examined the differential item functioning (DIF) contrast across gender and time spent gaming using Rasch analysis; three DIF items were found, namely Item 4, “Fail to control or cease gaming activities” concerning gender (DIF contrast=–0.55); item 4 concerning time spent online gaming per week (DIF contrast=–0.67); and item 9, “Jeopardize or lose an important thing because of gaming activity” concerning time spent online gaming per week (DIF contrast=.61).

### Reliability

Reliability of the IGDS9-SF was evaluated in four studies on four versions (n=2962). The studies investigated the test-retest reliability of the IGDS9-SF (ie, traditional Chinese [Hong Kong and Taiwan], Persian, and Turkish versions), and all demonstrated fair methodological quality. All studies also demonstrated a positive rating for the quality of statistical findings (Supplementary Table S2 in Multimedia Appendix 2), indicating the high reliability of the IGDS9-SF.

### Measurement Error

The above-mentioned four studies on four versions also examined the measurement errors in using the IGDS9-SF (n=2962; see Supplementary Table S3 in Multimedia Appendix 2). Of the four studies, three demonstrated doubtful methodological quality in traditional Chinese (Hong Kong and Taiwan) and Turkish versions, while the remaining study on the Persian version showed very good methodological quality. The standard error of measurement (SEM) was reported in two studies on the traditional Chinese (Hong Kong and Taiwan) and Persian versions. The SEM range of the four included studies was from 0.16 to 2.27. Given that only one study on the traditional Chinese (Hong Kong and Taiwan) versions reported the minimal important change (MIC) in the IGDS9-SF (smallest real difference=.44), the quality of this statistical finding remains indeterminate (Supplementary Table S3 in Multimedia Appendix 2).

### Criterion Validity

One study on the Turkish version evaluated criterion validity and showed very good methodological quality (Supplementary Table S3 in Multimedia Appendix 2). This study reported a strong correlation between the IGDS9-SF and the 27-item IGDS (*r*=.988) and demonstrated a positive rating for the quality of statistical findings.

### Concurrent Validity

Of the 21 studies, 11 using 11 versions evaluated the concurrent validity of the IGDS9-SF (Supplementary Table S4 in Multimedia Appendix 2). Very good methodological quality was noted in all 11 studies. Given the coefficient range (either the Pearson *r* correlation or the standardized regression coefficient) below 0.7, the rating for the quality of their statistical results was negative.

### Construct Validity (Convergent and Discriminant)

Of the 21 studies, 10 evaluated the convergent validity of the IGDS9-SF. Very good methodological quality was demonstrated in seven studies: traditional Chinese (Hong Kong and Taiwan), simplified Chinese, Spanish, Italian, European Portuguese, South American Portuguese, and Korean versions of the IGDS9-SF. Another three studies on the traditional Chinese (Hong Kong and Taiwan), English, and Turkish versions showed inadequate methodological quality. All 10 studies had a positive rating for the statistical quality (Supplementary Table S4 in Multimedia Appendix 2).

In addition, two studies evaluated the discriminative validity of the IGDS9-SF (Supplementary Table S5 in Multimedia Appendix 2). Both studies on the Turkish and Italian versions of the IGDS9-SF demonstrated very good methodological quality and had a positive rating for the statistical quality. Furthermore, two studies on the Italian and Turkish versions demonstrated significant gender differences on the IGDS9-SF score, with males having a higher level of IGD than females: *t*(451)=5.73, P=.001, *d*=.54 versus *t*(676.317)=6.61, P<.001. The study on the Italian version also showed significant age differences on the score, with young adults obtaining higher scores than old adults: *t*(648.267)=10.03 and P<.001.

### Other Psychometric Properties

#### Floor and Ceiling Effects

Floor and ceiling effects of the IGDS9-SF were reported in six studies on the English, traditional Chinese (Hong Kong), simplified Chinese, Persian, Turkish, and Malay versions. The study on the English version of the IGDS9-SF reported more gamers at floor-level scores (3.8%-5.6%) than at the ceiling level (0.2%-0.8%). The study on the Persian version of the IGDS9-SF reported a 1.8% ceiling effect and a 0.8% floor effect, whereas studies on the traditional Chinese (Hong Kong) and simplified Chinese versions reported no ceiling effect but a relatively high floor effect (21%-24.6%). Another study on the Turkish version did not report any ceiling effect of the IGDS9-SF but reported a 9% floor effect. A study on the Malay version of the IGDS9-SF reported a 1.7% ceiling effect and a 0.1% floor effect. Except for the studies on the traditional and simplified Chinese versions, the distribution of scoring on the IGDS9-SF in the related studies indicated acceptable floor and ceiling effects (ie, an effect less than 15%) [46].

#### IT

IT was reported in eight studies and ranged from 0.342 to 0.86 (Table 1). The items with the lowest IT in the Spanish version of the IGDS9-SF were item 7 (“Have you deceived any of your significant others because of the amount of your gaming activity?”) and item 8 (“Do you play to temporarily escape or relieve a negative mood?”) (IT=.47), while the lowest IT was in the traditional Chinese (Taiwan) version, which was found for item 5 (“Have you lost interest in previous hobbies because of your engagement with gaming?”) and item 7 (“Have you deceived any of your significant others because the amount of your gaming activity?”) (IT=.74).

Two studies on the traditional Chinese (Hong Kong) and Turkish versions also had the lowest IT correlation on item 7 (IT=.663-.68). Item 9 (“Have you jeopardized an important relationship, job, or educational or career opportunity because of your gaming activity?”) demonstrated the lowest IT in the South American Portuguese, traditional Chinese (Hong Kong), and simplified Chinese versions (IT=.342-.55), while the item with the lowest IT in the Persian version was item 4 (“Do you systematically fail when trying to control or cease your gaming activity?”) (IT=.54).

## Discussion

### Principal Findings

The quality of methodology and the quality of statistical findings for each psychometric property of the IGDS9-SF were evaluated for all 21 eligible studies. The evidence regarding the psychometric properties of the IGDS9-SF was summarized based on the existing evidence. In general, the IGDS9-SF demonstrated good internal consistency, although some items did not have satisfactory IT, especially items 7 (Spanish, Chinese, South American Portuguese, Turkish and Persian), 8 (Spanish, Chinese, and South American Portuguese), and 9 (Chinese, South American Portuguese, Turkish, and Persian). However, the IGDS9-SF has excellent criterion validity, as evidenced by the strong correlation with the 27-item IGDS [20]. Furthermore, the IGDS9-SF can distinguish different subgroups when assessing disordered gaming, with measurement invariance supported across both gender and age. In addition, the structure validity of the IGDS9-SF has been verified as a unidimensional structure among all 21 studies. Based on the aforementioned findings, the IGDS9-SF can best be used for clinicians to assess an individual’s IGD severity level.

### Internal Consistency and IT

All nine items of the IGDS9-SF demonstrated satisfactory IT (greater than 0.4) [22,23,25,30,32,48], except for items 7, 8, and 9 in one specific study [28]. Several plausible reasons for the unsatisfactory ITs are discussed here. The deception criterion (ie, item 7) may be influenced by the living status and external attitudes (eg, parents’ perception) on gaming behaviors [55,56] and therefore may be less associated with other IGDS9-SF items. According to previous findings, parents of a child living with them and indulging in excessive online gaming are usually aware of their child’s problematic gaming behavior and thus deception may not be a central IGD symptom [40]. This idea is supported by studies showing that the deception criterion is not associated with higher IGD severity [10]. If the severity of IGD in the population included is not high, a low IT may be reported. Furthermore, the deception criterion has shown low diagnostic accuracy in discriminating disordered gamers from nonproblematic gamers in previous studies due to the acceptability and accessibility of the internet [55] as well as the age of population investigated [40].

In relation to compromising occupation/education or a significant relationship due to the involvement in gaming (ie, item 9), a simpler criterion that only covers occupational/educational aspects is more appropriate than concurrently conflating relationships and occupational/educational loss [56]. For instance, the item may have greater clinical utility if it is split into two (ie, one reflecting the compromising of occupation/education and the other reflecting the compromising of significant relationships). Moreover, because the majority of the studies reviewed recruited individuals in full-time education, these particular participants might not feel that they have compromised their education, when responding to this item.

The behavior of escaping from adverse moods by gaming (ie, item 8) is reported as a criterion that is unable to differentiate between disordered and nondisordered gamers [57]. Internet use as a form of escapism could be considered as a coping strategy of a nondisordered gamer as much as that of a disordered gamer [58]. It is further supported by other studies showing that item 8 has the lowest specificity and diagnostic accuracy in discriminating disordered gamers and nondisordered gamers [20,40].

### Validity

The IGDS9-SF demonstrated a strong correlation with the 27-item IGDS [48], showing good criterion validity as expected. Like the IGDS9-SF, the 27-item IGDS was developed in accordance with the nine IGD criteria in the DSM-5. Therefore, the criterion validity of the IGDS9-SF is illustrated by the strong correlation observed with another psychometric test developed using the same DSM-5 criteria (ie, the 27-item IGDS) to measure IGD.

In addition to criterion validity, the concurrent validity of the IGDS9-SF was supported by the positive correlation between the IGDS9-SF score and the hours spent on online activities (eg, smartphone use, social media use, and gaming), with the correlations between the IGDS9-SF and gaming frequency being the most significant [19,22,24,25,28-31]. This implies that the frequency of gaming is positively associated with the severity of IGD.

In addition, a positive association was found between IGDS9-SF scores and three psychological symptoms (ie, depression, anxiety, and stress) [25,30]. This finding indicates that individuals with IGD might have a higher chance of suffering from mental distress, as evidenced by recent longitudinal findings [59]. One of the potential reasons underpinning this phenomenon might be the fact that excessive use of the internet can result in social withdrawal when an individual engages in gaming with features encouraging social disconnection (eg, features that increase social comparison and rumination [60,61]), which may lead to poorer psychological well-being among a minority of individuals [62]. This idea is also aligned with the time displacement hypothesis, which posits that the development and maintenance of social relationships require continuous commitment toward understanding, learning about other individuals, and communicating with them [63].

Although the IGDS9-SF is positively associated with other similar measures (eg, Mobile Phone-Related Experiences Questionnaire [CERM] and Online Gambling Disorder Questionnaire [OGD-Q]) [22-25,32,52], it only shows low-to-moderate correlation in most of the studies reviewed (*r*=.06-.440) [22-24,32]. The aforementioned measurements mainly focus on the conflicts and problems of smartphone use, social media use, and online gambling. These results show that the association of IGD with online gaming is greater than that with social media use or mobile phone use, which reaffirms that the IGDS9-SF has adequate psychometric properties when assessing the specific construct of IGD. One study showed an expected negative correlation between the IGDS9-SF and KIDSCREEN-27 [32]—an instrument assessing the quality of life—further supporting the notion that more severe IGD levels will likely lead to a poorer quality of life, such as physical or psychological well-being (eg, [59,64]).

### Factor Structure and Measurement Invariance

All 21 studies demonstrated the unidimensional structure of the IGDS9-SF and were supported by both confirmatory factor analysis and Rasch analysis. All factor loadings reported across the studies reviewed were satisfactory. Moreover, measurement invariance across gender [22,25,30,52] and age [32,52] was fully supported in most studies. However, measurement invariance across countries [26,50,51] and time spent gaming [30] was only partially supported. This phenomenon might be associated with the key cultural differences between the individualistic and collectivist countries. Research also suggests that cultural differences might be a plausible reason affecting the response patterns of psychometric instruments [65].

### Item Response Theory and Rasch Analysis

In addition to the most commonly used classical test theory (CTT) in the included studies, only two included studies used item response theory (or Rasch analysis) [31,49] for a better understanding of the psychometric properties of the IGD criteria beyond CTT. There is currently insufficient evidence on Rasch findings regarding the IGDS9-SF, given the scarcity of studies, and therefore future studies using Rasch-based models are required to provide further psychometric information about the IGDS9-SF.

### Strengths and Limitations

This systematic review had several strengths. First, a comprehensive search strategy was adopted to identify and evaluate potential studies. Second, the review evaluated studies with an accepted set of criteria, that is, both methodological quality (COSMIN) and statistical quality. Moreover, all 21 eligible studies included for evaluation in the systematic review had the strength of having good sample sizes (ie, N>100) based on the COSMIN risk-of-bias checklist. Therefore, this review took advantage of the good sample sizes of the studies evaluated to increase the accuracy in the summarized psychometric properties of the IGDS9-SF.

This review also had potential limitations. In the study selection stage, the language was limited to only English and each included study was assessed by only two authors (and these authors were not necessarily the same two for each paper). More specifically, although consensus was achieved in accordance with COSMIN guidelines, the authors who rated the quality of each study may have used slightly varied rating styles. Nevertheless, the COSMIN guidelines provided clear and concrete instructions for the evaluation of the studies, so the use of different authors in the study evaluation made it unlikely that this led to serious bias. Additionally, evaluation of the results was verified by the corresponding author, who is an expert psychometrician, which further minimized the possibility of evaluation bias. Some psychometric properties of the IGDS9-SF (eg, responsiveness) were not evaluated in any of the eligible studies. More specifically, responsiveness as an important psychometric property to understand whether an instrument is sensitive in detecting change was not carried out in any of the 21 studies. Further studies investigating responsiveness are therefore required. Finally, this systematic review did not use the Google Scholar database to supplement the literature search concerning the psychometric properties of the IGDS9-SF. Therefore, some studies may have been missed in this systematic review.

### Implications for Clinical Practice and Future Directions

The IGDS9-SF was designed both as a brief screening tool to assist clinicians in assessing IGD severity as well as for use in epidemiological studies [19]. Given that disordered gaming has been recognized by both the APA [13] and the World Health Organization [66], clinicians and other mental health professionals need to have a robust psychometric tool to assess IGD. Therefore, this review provides strong evidence enabling practitioners to better understand the psychometric features of the IGDS9-SF.

In sum, the IGDS9-SF is a time-efficient and psychometrically sound tool that can help clinicians screen for potential patients who may need more detailed clinical evaluation regarding their gaming behaviors. The use of the IGDS9-SF may also help in busy clinical settings by saving evaluation time in diagnosing and assessing IGD in patients. Indeed, a recent review of all screening instruments currently available for disordererd gaming (n=32) reported that the IGDS9-SF was among the best tools with regard to its psychometric properties when compared with all the others similar tools [21]. In terms of administration, the IGDS9-SF takes only a few minutes for individuals to complete it by themselves. This can substantially reduce the time for clinicians to diagnose and assess IGD symptoms. To this end, clinical studies have suggested adopting a cutoff of 32 points when diagnosing between disordered and nondisordered gamers [67]. Moreover, due to the robust psychometric properties reported across 15 language versions, the IGDS9-SF can also be used in worldwide epidemiological studies examining IGD [40]. Given its brevity and multilanguage capability, the IGDS9-SF can be used in collecting valid and reliable data concerning IGD symptoms in a practical and efficient way that minimizes survey fatigue and can also be used for cross-cultural comparisons in different countries to help advance our understanding of IGD.

### Conclusion

This systematic review summarized and reviewed evidence from various populations regarding the IGDS9-SF with regard to its structural validity, internal consistency, cross-cultural validity/measurement invariance, reliability, measurement error, criterion validity, convergent validity, and discriminative or known-group validity. Overall, there was strong evidence demonstrating that the IGDS9-SF has good internal consistency and excellent criterion validity for wide-ranging populations. Responsiveness and reliability as two important psychometric properties of the IGDS9-SF require more evidence because few studies have evaluated them. Regarding the psychometric evidence of different language versions, it was found that all versions of the IGDS9-SF present strong psychometric properties, except for the concurrent validity of the traditional Chinese (Hong Kong and Taiwan), simplified Chinese, Polish, and Persian versions. As such, the psychometric properties of the IGDS9-SF are robust, and the findings regarding its cross-cultural psychometric features in different language versions have been further clarified. Therefore, the IGDS9-SF can be used widely within clinical and research settings.

## Appendix

Multimedia Appendix 1 Database search.

Multimedia Appendix 2 Results of studies on measurement properties.

## Abbreviations

APA: American Psychiatric Association
CERM: Mobile Phone-Related Experiences Questionnaire
COSMIN: Consensus-based Standards for the selection of health status Measurement Instrument
CTT: classical test theory
DIF: differential item functioning
DSM-5: *Diagnostic and Statistical Manual of Mental Disorders, 5th Edition*
ESL: Electronic Sports League
ICD-11: *International Classification of Diseases*, 11th revision
IGD: internet gaming disorder
IGDS9-SF: Internet Gaming Disorder Scale–Short-Form
IGDT: Internet Gaming Disorder Test
IT: item-total correlation
MIC: minimal important change
MMO: massively multiplayer online
MOBA: multiplayer online battle arena
OGD-Q: Online Gambling Disorder Questionnaire
PROM: patient-reported outcome measure
RMSEA: root-mean-square error of approximation
SEM: standard error of measurement
SRMR: standardized root-mean-square residual
