# Supplementary material for: Psychometric Properties of the Internet Gaming Disorder Scale–Short-Form (IGDS9-SF): Systematic Review
Source: J Med Internet Res. 2021 Oct 18;23(10):e26821. doi: 10.2196/26821 (PMC8561410; doi:10.2196/26821)
Supplement: Multimedia Appendix 1 [file jmir_v23i10e26821_app1.docx]

Appendix A. Database search

The following terms were used in the literature search on the seven databases.

1. Embase

| Search terms | ('IGD9-SF' OR 'IGDS-SF9' OR 'IGD-SF' OR 'internet gaming disorder scale-short-form' OR 'internet gaming disorder scale short form' OR 'internet gaming disorder scale - 9-item short form' OR 'nine-item internet gaming disorder scale - short form') AND [1-1-1998]/sd NOT [10-10-2020]/sd |
| --- | --- |

1. Medline

| Boolen / Phrase | IGDS9-SF OR IGDS-SF9 OR IGD-SF OR Internet Gaming Disorder Scale-Short-form OR Internet Gaming Disorder Scale Short form OR Internet Gaming Disorder Scale - 9-Item Short Form OR Nine-item Internet Gaming Disorder Scale - Short Form |
| --- | --- |
| Limiters | Date of Publication: 19980101-20201010 |

1. PsycINFO

| Search terms | IGDS9-SF OR IGDS-SF9 OR IGD-SF OR (Internet Gaming Disorder Scale-Short-form) OR (Internet Gaming Disorder Scale Short form) OR (Internet Gaming Disorder Scale - 9-Item Short Form) OR (Nine-item Internet Gaming Disorder Scale - Short Form) |
| --- | --- |
| Limiters | Date of Publication: From January 01 1998 to October 10 2020 |

1. PubMed

| Search terms | ('IGDS9-SF' OR 'IGDS-SF9' OR 'IGD-SF' OR 'Internet Gaming Disorder Scale-Short-form' OR 'Internet Gaming Disorder Scale Short form' OR 'Internet Gaming Disorder Scale - 9-Item Short Form' OR 'Nine-item Internet Gaming Disorder Scale - Short Form') AND (("1998/01/01"[Date - Publication] : "2020/10/10"[Date - Publication])) |
| --- | --- |

1. ScienceDirect

| Search terms | 'IGDS9-SF' OR 'IGDS-SF9' OR 'IGD-SF' OR 'Internet Gaming Disorder Scale-Short-form' OR 'Internet Gaming Disorder Scale Short form' OR 'Internet Gaming Disorder Scale - 9-Item Short Form' OR 'Nine-item Internet Gaming Disorder Scale - Short Form' |
| --- | --- |
| Year | 1998-2020 |

1. Scopus

| Search terms | All ("IGDS9-SF" OR "IGDS-SF9 " OR "IGD-SF" OR "Internet Gaming Disorder Scale-Short-form" OR "Internet Gaming Disorder Scale Short form" OR "Internet Gaming Disorder Scale - 9-Item Short Form" OR "Nine-item Internet Gaming Disorder Scale - Short Form") AND (PUBYEAR > 1997 AND PUBYEAR < 2021) |
| --- | --- |

1. Web of Science

| Search terms | ALL FIELDS: ('IGDS9-SF' OR 'IGDS-SF9' OR 'IGD-SF' OR 'Internet Gaming Disorder Scale-Short-form' OR 'Internet Gaming Disorder Scale Short form' OR 'Internet Gaming Disorder Scale - 9-Item Short Form' OR 'Nine-item Internet Gaming Disorder Scale - Short Form') *AND* YEAR PUBLISHED: (1998-2020) |
| --- | --- |

ScienceDirect and Web of Science databases only allow search of year of publication instead of date of publication. Articles published before 1 January, 1998 were removed manually with the use of endnote, if necessary.
