# Supplementary material for: Psychometric Properties of the Internet Gaming Disorder Scale–Short-Form (IGDS9-SF): Systematic Review
Source: J Med Internet Res. 2021 Oct 18;23(10):e26821. doi: 10.2196/26821 (PMC8561410; doi:10.2196/26821)
Supplement: Multimedia Appendix 2 [file jmir_v23i10e26821_app2.docx]

Appendix B. Table S1. Results of studies on measurement properties (structural validity and internal consistency)

| **PROM (ref)** | **Country (language) in which the questionnaire was evaluated** | **Structural validity** | | | **Internal consistency** | | |
| --- | --- | --- | --- | --- | --- | --- | --- |
|  |  | **N** | **Meth qual*** | **Result (rating^#^)** | **N** | **Meth qual*** | **Result (rating^#^)** |
| Chen et al., 2020 [24] | Hong Kong (Traditional Chinese) | 304 | V | CTT (CFA): CFI 0.992, RMSEA 0.0735, SRMR 0.0475 (+) | 304 | V | Cronbach’s alpha = 0.916 at baseline, 0.920 at follow-up (+) |
|  | Taiwan (Traditional Chinese) | 336 | V |  | 336 | V |  |
| Beranuy et al., 2020 [32] | Navarre (Spanish) | 535 | V | CTT (CFA): CFI 0.995, RMSEA 0.019, SRMR 0.035 (+) | 535 | V | Cronbach’s alpha = 0.85 (+) |
| de Palo et al., 2019 [26] | Albania (Albanian) | 228 | V | CTT (CFA): CFI 0.907, RMSEA 0.142 (?) | 228 | V | Cronbach’s alpha = 0.810 (+) |
|  | USA (English) | 237 | V | CTT (CFA): CFI 0.977, RMSEA 0.078 (?) | 237 | V | Cronbach’s alpha = 0.841 (+) |
|  | UK (English) | 275 | V | CTT (CFA): CFI 0.973, RMSEA 0.104 (?) | 275 | V | Cronbach’s alpha = 0.870 (+) |
|  | Italy (Italian) | 671 | V | CTT (CFA): CFI 0.997, RMSEA 0.104 (?) | 671 | V | Cronbach’s alpha = 0.963 (+) |
| Evren et al., 2017 [48] | Turkey (Turkish) | 457 | I | CTT (CFA): CFI 0.987, RMSEA 0.064 (?) | 457 | V | Cronbach’s alpha = 0.931 (+) |
| Gomez et al., 2019 [49] | USA (English) | 868 | V | CTT (CFA): CFI 0.991, RMSEA 0.021 (?) | 868 | V | Cronbach’s alpha = 0.91 (+) |
| Leung et al., 2020 [22] | Taiwan (Traditional Chinese) | 336 | V | CTT (CFA): CFI 0.999, RMSEA 0.016 (?) | 336 | V | Cronbach’s alpha = 0.94 (+) |
|  | Hong Kong (Traditional Chinese) | 306 | V | CTT (CFA): CFI 1.000, RMSEA 0.000 (?) | 306 | V | Cronbach’s alpha = 0.93 (+) |
| Monacis et al., 2016 [52] | Italy (Italian) | 687 | V | CTT (CFA): CFI 0.970, RMSEA 0.072, SRMR 0.02 (+) | 687 | V | Cronbach’s alpha = 0.96 (+) |
| Pontes & Griffiths, 2015 [19] | 52 different English-  speaking countries (English) | 1060 | V | CTT (CFA): CFI 0.964, RMSEA 0.054, SRMR 0.034 (+) | 1060 | V | Cronbach’s alpha = 0.88 (+) |
| Pontes & Griffiths, 2016 [27] | Portugal (European Portuguese) | 509 | V | CTT (CFA): CFI 0.974, RMSEA 0.039 (?) | 509 | V | Cronbach’s alpha = 0.87 (+) |
| Pontes et al., 2017 [50] | USA (English) | 405 | V | CTT (CFA): CFI 0.94, RMSEA 0.08, SRMR 0.04 (+) | 405 | V | Cronbach’s alpha = 0.91 (+) |
|  | India (English) | 336 | V | CTT (CFA): CFI 0.97, RMSEA 0.05, SRMR 0.03 (+) | 336 | V | Cronbach’s alpha = 0.89 (+) |
|  | UK (English) | 272 | V | CTT (CFA): CFI 0.92, RMSEA 0.07, SRMR 0.05 (+) | 272 | V | Cronbach’s alpha = 0.88 (+) |
| Pontes et al., 2016 [29] | Slovenia (Slovenian) | 1071 | V | CTT (CFA): CFI 0.99, RMSEA 0.030, SRMR 0.022 (+) | 1071 | V | Cronbach’s alpha = 0.93 (+) |
| Schivinski et al., 2018 [31] | Poland (Polish) | 3222 | V | CTT (CFA): CFI 0.968, RMSEA 0.043, SRMR 0.025 (+) | 3222 | V | Cronbach’s alpha = 0.82 (+) |
| Severo et al., 2020 [28] | Brazil (South American Portuguese) | 555 | V | CTT (CFA): CFI 0.97, RMSEA 0.051, SRMR 0,029 (+) | 555 | V | Cronbach’s alpha = 0.82 (+) |
| Stavropoulos et al., 2019 [54] | USA (English) | 120 | V | CTT (CFA) (TP1): CFI 0.99, TLI 0.98, RMSEA 0.093 (?)  CTT (CFA) (TP2): CFI 0.97, TLI 0.96, RMSEA 0.148 (?) | 120 | V | Cronbach’s alpha (TP1) = 0.91 (+)  Cronbach’s aplha (TP2) = 0.92 (+) |
|  | Australia (English) | 61 | A | CTT (CFA) (TP1): CFI 0.99, RMSEA 0.039, TLI 0.98 (?)  CTT (CFA) (TP2): CFI 0.93, RMSEA 0.094, TLI 0.9 (?) | 61 | V | Cronbach’s alpha (TP1) = 0.87 (+)  Cronbach’s alpha (TP2) = 0.89 (+) |
| Wu et al., 2017 [30] | Iran (Persian) | 2363 | V | CTT (CFA): CFI 0.990, RMSEA 0.061, SRMR 0.049 (+) | 2363 | V | CTT: Cronbach’s alpha = 0.9 (+)  Rasch: Person separation reliability = 0.86 (+) |
| Yam et al., 2019 [23] | Hong Kong (Traditional Chinese) | 307 | V | CTT (CFA): CFI 0.969, RMSEA 0.077, SRMR 0.041 (+) | 307 | V | Cronbach’s alpha = 0.903 (+) |
| Stavropoulos et al., 2018 [51] | Australia (English) | 171 | V | CTT (CFA): SRMR 0.051 (+) | 171 | V | Cronbach’s alpha = 0.90 (+) |
|  | USA (English) | 463 | V | CTT (CFA): SRMR 0.048 (+) | 463 | V | Cronbach’s alpha = 0.91 (+) |
|  | UK (English) | 281 | V | CTT (CFA): SRMR 0.048 (+) | 281 | V | Cronbach’s alpha = 0.89 (+) |
| Aricak et al., 2018 [35] | Turkey (Turkish) | 455 | V | CTT (CFA): CFI 0.90, TLI 0.87, RMSEA 0.09 (?) | 455 | V | Cronbach’s alpha = 0.82 (+) |
| Chen et al., 2020 [25] | Mainland China (Simplified Chinese) | 1108 | V | CTT (CFA): CFI 0.98, TLI 0.98, RMSEA 0.076, SRMR 0.067 (+) | 1108 | V | Cronbach’s alpha = 0.84 (+) |
| Kim & Ko, 2020 [39] | Korea (Korean) | 594 | V | CTT (CFA): CFI 0.956, TLI 0.926, RMSEA 0.071 (?) | 594 | V | Cronbach’s alpha = 0.86 (+) |
| T’ng & Pau, 2020 [38] | Malaysia (Bahasa Malasia) | 1050 | V | CTT (CFA) (Sample 1): CFI 0.955, RMSEA 0.059, SRMR 0.042 (+)  CTT (CFA) (Sample 2): CFI 0.969, RMSEA 0.052, SRMR 0.035 (+) | 1050 | V | Cronbach’s alpha = 0.81 (+) |
| **Pooled or summary result**  **(overall rating)** | | **19049** | **I** | **One factor (+)** | **19049** | **V** | **0.810 - 0.963 (+)** |

NA = Not available, CTT = Classical Test Theory, CFA =confirmatory factor analysis, CFI =comparative fit index, TLI = Tucker-Lewis index, RMSEA = root mean square of approximation, SRMR = standardized root mean square residual

***COSMIN score after removing the sample size item from the rating: V, very good; A, adequate; D, doubtful; I, inadequate; N, not applicable. ^#^Quality score of the measurement property: +, sufficient; -, insufficient; ?, indeterminate**

Table S2. Results of studies on measurement properties (cross-cultural validity and reliability)

| **PROM (ref)** | **Country (language) in which the questionnaire was evaluated** | **Cross-cultural validity** | | | **Reliability** | | |
| --- | --- | --- | --- | --- | --- | --- | --- |
|  |  | **N** | **Meth qual*** | **Result (rating^#^)** | **N** | **Meth qual*** | **Result (rating^#^)** |
| Chen et al., 2020 [24] | Hong Kong (Traditional Chinese) | NA | NA | NA | 156 | D | ICC = 0.94 (+) |
|  | Taiwan (Traditional Chinese) | NA | NA | NA | 118 | D |  |
| Beranuy et al., 2020 [32] | Navarre (Spanish) | 535 | D | Gender (+)  ∆CFI = -0.013 to -0.003 (partial invariance)  note: male fits better than female  Age (+)  ∆CFI = -0.005 to -0.004  (full invariance) | NA | NA | NA |
| de Palo et al., 2019 [26] | Albania (Albanian) | 228 | D | Country (+)  ΔCFI = 0.000 to 0.010 (partial invariance) | NA | NA | NA |
|  | USA (English) | 237 | D |  | NA | NA | NA |
|  | UK (English) | 275 | D |  | NA | NA | NA |
|  | Italy (Italian) | 671 | D |  | NA | NA | NA |
| Evren et al., 2017 [48] | Turkey (Turkish) | NA | NA | NA | 261 | D | Pearson product–moment correlation 0.756 (?) |
| Gomez et al., 2019 [49] | USA (English) | NA | NA | NA | NA | NA | NA |
| Leung et al., 2020 [22] | Taiwan (Traditional Chinese) | 336 | I | Country (+)  ΔCFI = 0.000 to 0.002 (full invariance) | NA | NA | NA |
|  | Hong Kong (Traditional Chinese) | 306 | I |  | NA | NA | NA |
| Monacis et al., 2016 [52] | Italy (Italian) | 687 | D | Gender (+)  ΔCFI = 0.004 to 0.008 (full invariance)  Age (+)  ΔCFI = 0.001 to 0.003 (full invariance) | NA | NA | NA |
| Pontes & Griffiths, 2015 [19] | 52 different English- speaking countries (English) | NA | NA | NA | NA | NA | NA |
| Pontes & Griffiths, 2016 [27] | Portugal (European Portuguese) | NA | NA | NA | NA | NA | NA |
| Pontes et al., 2017 [50] | USA (English) | 405 | A | Countries (-)  ΔCFI = -0.01 to -0.05 (partial invariance) | NA | NA | NA |
|  | India (English) | 336 | A |  | NA | NA | NA |
|  | UK (English) | 272 | A |  | NA | NA | NA |
| Pontes et al., 2016 [29] | Slovenia (Slovenian) | NA | NA | NA | NA | NA | NA |
| Schivinski et al., 2018 [31] | Poland (Polish) | NA | NA | NA | NA | NA | NA |
| Severo et al., 2020 [28] | Brazil (South American Portuguese) | NA | NA | NA | NA | NA | NA |
| Stavropoulos et al., 2019 [54] | USA (English) | NA | NA | NA | NA | NA | NA |
|  | Australia (English) | NA | NA | NA | NA | NA | NA |
| Wu et al., 2017 [30] | Iran (Persian) | 2363 | D | Gender (+)  ΔCFI = -0.002 (full invariance)  Hour spent online gaming per week (+)  ΔCFI = -0.009 to -0.006 (partial invariance) | 2363 | D | Pearson's correlation 0.87 (?) |
| Yam et al., 2019 [23] | Hong Kong (Traditional Chinese) | NA | NA | NA | NA | NA | NA |
| Stavropoulos et al., 2018 [51] | Australia (English) | 171 | D | Country (-)  ΔCFI = -0.009 to -0.023 (partial invariance) | NA | NA | NA |
|  | USA (English) | 463 | D |  | NA | NA | NA |
|  | UK (English) | 281 | D |  | NA | NA | NA |
| Aricak et al., 2018 [35] | Turkey (Turkish) | NA | NA | NA | 64 | D | Pearson product-moment correlation coefficient 0.78 (?) |
| Chen et al., 2020 [25] | Mainland China (Simplified Chinese) | 1099 | D | Gender (+)  ΔCFI = 0.000  (full invariance) | NA | NA | NA |
| Kim & Ko, 2020 [39] | Korea (Korean) | NA | NA | NA | NA | NA | NA |
| T’ng & Pau, 2020 [38] | Malaysia (Bahasa Malaysia) | 1050 | D | Gender (+)  ΔCFI = 0.001-0.002  (full invariance) | NA | NA | NA |
| **Pooled or summary result**  **(overall rating)** | | **7352** | **I** | **1. Age (+) full invariance**  **2. Gender (?) partial invariance**  **3. Time on gaming (+) partial invariance**  **4. Country (-) partial invariance** | **2962** | **D** | **ICC = 0.94**  **Pearson correlation = 0.756 to 0.87** |

NA = Not available, ΔCFI = difference between the comparative fit index, ICC = intraclass correlation coefficient

***COSMIN score after removing the sample size item from the rating: V, very good; A, adequate; D, doubtful; I, inadequate; N, not applicable. ^#^Quality score of the measurement property: +, sufficient; -, insufficient; ?, indeterminate**

Table S3. Results of studies on measurement properties (measurement error and criterion validity)

| **PROM (ref)** | **Country (language) in which the questionnaire was evaluated** | **Measurement error** | | | **Criterion validity** | | |
| --- | --- | --- | --- | --- | --- | --- | --- |
|  |  | **N** | **Meth qual*** | **Result (rating^#^)** | **N** | **Meth qual*** | **Result (rating^#^)** |
| Chen et al., 2020 [24] | Hong Kong (Traditional Chinese) | 156 | D | SEM 0.16 < SRD 0.44 (+) | NA | NA | NA |
|  | Taiwan (Traditional Chinese) | 118 | D |  | NA | NA | NA |
| Beranuy et al., 2020 [32] | Navarre (Spanish) | NA | NA | NA | NA | NA | NA |
| de Palo et al., 2019 [26] | Albania (Albanian) | NA | NA | NA | NA | NA | NA |
|  | USA (English) | NA | NA | NA | NA | NA | NA |
|  | UK (English) | NA | NA | NA | NA | NA | NA |
|  | Italy (Italian) | NA | NA | NA | NA | NA | NA |
| Evren et al., 2017 [48] | Turkey (Turkish) | 261 | D | No SEM reported (?) | 457 | V | 27 item-IGDS (r = 0.988) (+) |
| Gomez et al., 2019 [49] | USA (English) | NA | NA | NA | NA | NA | NA |
| Leung et al., 2020 [22] | Taiwan (Traditional Chinese) | NA | NA | NA | NA | NA | NA |
|  | Hong Kong (Traditional Chinese) | NA | NA | NA | NA | NA | NA |
| Monacis et al., 2016 [52] | Italy (Italian) | NA | NA | NA | NA | NA | NA |
| Pontes & Griffiths, 2015 [19] | 52 different English- speaking countries (English) | NA | NA | NA | NA | NA | NA |
| Pontes & Griffiths, 2016 [27] | Portugal (European Portuguese) | NA | NA | NA | NA | NA | NA |
| Pontes et al., 2017 [50] | USA (English) | NA | NA | NA | NA | NA | NA |
|  | India (English) | NA | NA | NA | NA | NA | NA |
|  | UK (English) | NA | NA | NA | NA | NA | NA |
| Pontes et al., 2016 [29] | Slovenia (Slovenian) | NA | NA | NA | NA | NA | NA |
| Schivinski et al., 2018 [31] | Poland (Polish) | NA | NA | NA | NA | NA | NA |
| Severo et al., 2020 [28] | Brazil (South American Portuguese) | NA | NA | NA | NA | NA | NA |
| Stavropoulos et al., 2019 [54] | USA (English) | NA | NA | NA | NA | NA | NA |
|  | Australia (English) | NA | NA | NA | NA | NA | NA |
| Wu et al., 2017 [30] | Iran (Persian) | 2363 | V | SEM 2.27 (?) | NA | NA | NA |
| Yam et al., 2019 [23] | Hong Kong (Traditional Chinese) | NA | NA | NA | NA | NA | NA |
| Stavropoulos et al., 2018 [51] | Australia (English) | NA | NA | NA | NA | NA | NA |
|  | USA (English) | NA | NA | NA | NA | NA | NA |
|  | UK (English) | NA | NA | NA | NA | NA | NA |
| Aricak et al., 2018 [35] | Turkey (Turkish) | 64 | D | No SEM reported (?) | NA | NA | NA |
| Chen et al., 2020 [25] | Mainland China (Simplified Chinese) | NA | NA | NA | NA | NA | NA |
| Kim & Ko, 2020 [39] | Korea (Korean) | NA | NA | NA | NA | NA | NA |
| T’ng & Pau, 2020 [38] | Malaysia (Bahasa Malaysia) | NA | NA | NA | NA | NA | NA |
| **Pooled or summary result**  **(overall rating)** | | **2962** | **D** | **0.16 to 2.27 (?)** | **457** | **V** | **r = 0.988 (+)** |

NA = Not available, 27-item IGDS = 27-item Internet Gaming Disorder Scale, SEM = standard error measurement, SRD = difference between the comparative fit index

***COSMIN score after removing the sample size item from the rating: V, very good; A, adequate; D, doubtful; I, inadequate; N, not applicable. ^#^Quality score of the measurement property: +, sufficient; -, insufficient; ?, indeterminate**

Table S4. Results of studies on measurement properties (concurrent and convergent validity)

| **PROM (ref)** | **Country (language) in which the questionnaire was evaluated** | **Concurrent validity** | | | **Convergent validity** | | |
| --- | --- | --- | --- | --- | --- | --- | --- |
|  |  | **N** | **Meth qual*** | **Result (rating^#^)** | **N** | **Meth qual*** | **Result (rating^#^)** |
| Chen et al., 2020 [24] | Hong Kong (Traditional Chinese) | 304 | V | 1. Time on smartphone   r = 0.00   1. Time on social media   r = -0.10   1. Time on gaming   rs = 0.28  (-) | 304 | V | 1. Baseline IGDS-SF9/ Follow up SABAS r = 0.26 2. Baseline IGDS-SF9/ Follow up BSMAS r = 0.10 3. Follow-up IGDS-SF9/ Follow up SABAS r = 0.26 4. Follow-up IGDS-SF9/ Follow up BSMAS r = 0.06 |
|  | Taiwan (Traditional Chinese) | 336 | V |  | 336 | V |  |
| Beranuy et al., 2020 [32] | Navarre (Spanish) | NA | NA | NA | 101 | V | 1. IGDS9-SF/OGD-Q r = 0.440, p < 0.001 2. IGDS9-SF/CERM r = 0.553, p = 0.001 3. IGDS9-SF/KIDSCREEN-27  - Physical well-being r = -0.164, p = 0.001 - Psychological well-being r = -0.315, p = 0.001 - Autonomy and relationship with parents r = -0.167, p = 0.001 - Peers and social support r = -0.257, p = 0.001 - School environment r = -0.176, p = 0.001 |
| de Palo et al., 2019 [26] | Albania (Albanian) | NA | NA | NA | NA | NA | NA |
|  | USA (English) | NA | NA | NA | NA | NA | NA |
|  | UK (English) | NA | NA | NA | NA | NA | NA |
|  | Italy (Italian) | NA | NA | NA | NA | NA | NA |
| Evren et al., 2017 [48] | Turkey (Turkish) | 457 | V | Time daily spend on the Internet rs = 0.556 (-) | NA | NA | NA |
| Gomez et al., 2019 [49] | USA (English) | NA | NA | NA | NA | NA | NA |
| Leung et al., 2020 [22] | Taiwan (Traditional Chinese) | 336 | V | 1. Time on social media   r = -0.111   1. Time on smartphone   r = -0.038   1. Time on gaming   r = 0.430  (-) | 336 | I | 1. IGD9-SF/BSMAS r = 0.101 2. IGD9-SF/SABAS r = 0.356 |
|  | Hong Kong (Traditional Chinese) | 306 | V |  | 306 | I |  |
| Monacis et al., 2016 [52] | Italy (Italian) | NA | NA | NA | 687 | V | 1. IGDS9-SF/ BSMAS r = 0.764 2. IGDS9-SF/ GAS r = 0.809 3. IGDS9-SF/ IAT r = 0.827 |
| Pontes & Griffiths, 2015 [19] | 52 different English- speaking countries (English) | 1060 | V | IGDS-SF9/ Weekly gameplay r = 0.319 (-) | 1060 | I | IGDS-SF9/ IGD-20 Test r = 0.816 |
| Pontes & Griffiths, 2016 [27] | Portugal (European Portuguese) | NA | NA | NA | NA | NA | NA |
| Pontes et al., 2017 [50] | USA (English) | NA | NA | NA | NA | NA | NA |
|  | India (English) | NA | NA | NA | NA | NA | NA |
|  | UK (English) | NA | NA | NA | NA | NA | NA |
| Pontes et al., 2016 [29] | Slovenia (Slovenian) | 1071 | V | 1. Time spent in gaming on weekdays r = 0.47 2. Time spent in gaming on weekends r = 0.52   (-) | NA | NA | NA |
| Schivinski et al., 2018 [31] | Poland (Polish) | 3377 | V | 1. Hours/Weekdays   β =0.085   1. Hours/Weekend   β =0.364   1. Hours/Session   β =0.093  (-) | NA | NA | NA |
| Severo et al., 2020 [28] | Brazil (South American Portuguese) | 555 | V | IGDS-SF9/ weekly gaming hours r = 0.461 (-) | 555 | V | IGDS-SF9/ GAS scores r = 0.820 |
| Stavropoulos et al., 2019 [54] | USA (English) | NA | NA | NA | NA | NA | NA |
|  | Australia (English) | NA | NA | NA | NA | NA | NA |
| Wu et al., 2017 [30] | Iran (Persian) | 2363 | V | 1. IGDS-SF9/ Depression   β = 0.139, ΔR^2^ = 0.02   1. IGDS-SF9/ Anxiety   β = 0.148, ΔR^2^ = 0.02   1. IGDS-SF9/ Stress   β = 0.103, ΔR^2^ = 0.01   1. IGDS-SF9/ Hours spent online gaming per week   β = 0.663, ΔR^2^ = 0.39  (-) | NA | NA | NA |
| Yam et al., 2019 [23] | Hong Kong (Traditional Chinese) | NA | NA | NA | 307 | V | 1. IGDS-SF9/BSMAS r = 0.22 2. IGDS-SF9/SABAS r = 0.35 |
| Stavropoulos et al., 2018 [51] | Australia (English) | NA | NA | NA | NA | NA | NA |
|  | USA (English) | NA | NA | NA | NA | NA | NA |
|  | UK (English) | NA | NA | NA | NA | NA | NA |
| Aricak et al., 2018 [35] | Turkey (Turkish) | NA | NA | NA | 455 | V | IGDS-SF9/IAS r = 0.57 |
| Chen et al., 2020 [25] | Mainland China (Simplified Chinese) | 1108 | V | 1. Depression r=0.55 2. Anxiety r=0.47 3. Stress r=0.52 4. Time on smartphone r=0.29 5. Time on social media r=0.16 6. Time on gaming r=0.20 (-) | 1108 | V | 1. IGDS-SF9/BSMAS r=0.55 2. IGDS-SF9/SABAS r=0.69   (-) |
| Kim & Ko, 2020 [39] | Korea (Korean) | 594 | V | 1. Weekly gaming hour r=0.31 2. Weekly gaming day r=0.32 3. Adjacent personality and affective variables, impulsivity  r=0.29 4. Loneliness r=0.36 5. Self-esteem  r=-0.31   (-) | 594 | V | 1. IGDS9-SF/IGD-20 Test r=0.85 2. IGDS9-SF/RSES r=0.31 3. IGDS9-SF/BIS r=0.29 4. IGDS9-SF/UCLA LS r=0.36 |
| T’ng & Pau, 2020 [38] | Malaysia (Bahasa Malaysia) | 1050 | V | 1. Gaming frequency per week r=0.09 2. Gaming duration per day  r=0.28 3. Gaming experience in years r=0.07 4. Starting age of playing online games  r=0.06   (-) | NA | NA | NA |
| **Pooled or summary result (overall rating)** | | 12323 | V | Absolute r = 0.00 to 0.556  Absolute β = 0.103 to 0.663 | 6149 | V | Absolute r = 0.06 to 0.827 |

NA = Not available, BSMAS= Bergen Social Media Addiction Scale, CERM = Cuestionario de Experiencias Relacionadas con el teléfono móvil [Mobile Phone-Related Experiences Questionnaire], GAS=Gaming Addiction Scale, IAS=Internet Addiction Scale, IAT=Internet Addiction Test, IGD=Internet Gaming Disorder, OGD-Q = Online Gambling Disorder Questionnaire, SABAS=Smartphone Application-Based Addiction Scale, RSES=Rosenberg Self-Esteem Scale, BIS=Barratt Impulsiveness Scale, UCLA LS=UCLA Loneliness Scael, rs = spearman correlation, r = Pearson’s correlations coefficients, β = standardized coefficient, p = p-value, ΔR^2^ = R^2^ of the criterion plus confounders minus the R^2^ of the confounders

***COSMIN score after removing the sample size item from the rating: V, very good; A, adequate; D, doubtful; I, inadequate; N, not applicable. ^#^Quality score of the measurement property: +, sufficient; -, insufficient; ?, indeterminate**

Table S5. Results of studies on measurement properties (discriminative validity)

| **PROM (ref)** | **Country (language) in which the questionnaire was evaluated** | **Discriminative validity** | | |
| --- | --- | --- | --- | --- |
|  |  | **N** | **Meth qual*** | **Result (rating^#^)** |
| Chen et al., 2020 [24] | Hong Kong (Traditional Chinese) | NA | NA | NA |
|  | Taiwan (Traditional Chinese) | NA | NA | NA |
| Beranuy et al., 2020 [32] | Navarre (Spanish) | NA | NA | NA |
| de Palo et al., 2019 [26] | Albania (Albanian) | NA | NA | NA |
|  | USA (English) | NA | NA | NA |
|  | UK (English) | NA | NA | NA |
|  | Italy (Italian) | NA | NA | NA |
| Evren et al., 2017 [48] | Turkey (Turkish) | NA | NA | NA |
| Gomez et al., 2019 [49] | USA (English) | NA | NA | NA |
| Leung et al., 2020 [22] | Taiwan (Traditional Chinese) | NA | NA | NA |
|  | Hong Kong (Traditional Chinese) | NA | NA | NA |
| Monacis et al., 2016 [52] | Italy (Italian) | 687 | V | 1. Sex difference: t(676.317) = 6.61, p <0.001 2. Age difference: t(648.267) = 10.03, p <0.001 |
| Pontes & Griffiths, 2015 [19] | 52 different English-speaking countries (English) | NA | NA | NA |
| Pontes & Griffiths, 2016 [27] | Portugal (European Portuguese) | NA | NA | NA |
| Pontes et al., 2017 [50] | USA (English) | NA | NA | NA |
|  | India (English) | NA | NA | NA |
|  | UK (English) | NA | NA | NA |
| Pontes et al., 2016 [29] | Slovenia (Slovenian) | NA | NA | NA |
| Schivinski et al., 2018 [31] | Poland (Polish) | NA | NA | NA |
| Severo et al., 2020 [28] | Brazil (South American Portuguese) | NA | NA | NA |
| Stavropoulos et al., 2019 [54] | USA (English) | NA | NA | NA |
|  | Australia (English) | NA | NA | NA |
| Wu et al., 2017 [30] | Iran (Persian) | NA | NA | NA |
| Yam et al., 2019 [23] | Hong Kong (Traditional Chinese) | NA | NA | NA |
| Stavropoulos et al., 2018 [51] | Australia (English) | NA | NA | NA |
|  | USA (English) | NA | NA | NA |
|  | UK (English) | NA | NA | NA |
| Aricak et al., 2018 [35] | Turkey (Turkish) | 455 | V | Sex difference: t(451) = 5.73, p = 0.001, d = 0.54 |
| Chen et al., 2020 [25] | Mainland China (Simplified Chinese) | NA | NA | NA |
| Kim & Ko, 2020 [39] | Korea (Korean) | NA | NA | NA |
| T’ng & Pau, 2020 [38] | Malaysia (Bahasa Malaysia) | NA | NA | NA |
| **Pooled or summary result (overall rating)** | | 1142 | D | Significant difference found in age and gender |

NA = Not available, p = p-value

***COSMIN score after removing the sample size item from the rating: V, very good; A, adequate; D, doubtful; I, inadequate; N, not applicable. ^#^Quality score of the measurement property: +, sufficient; -, insufficient; ?, indeterminate**
